# Zinc in Preventing the Progression of pre-Diabetes (ZIPPeD Study) – study protocol for a randomised placebo-controlled trial in Australia

**DOI:** 10.1186/s13063-019-3317-4

**Published:** 2019-04-15

**Authors:** Roseanne Peel, Alexis Hure, John Wiggers, Mark McEvoy, Elizabeth Holliday, Andrew Searles, Penny Reeves, Priyanga Ranasinghe, Ranil Jayawardena, Samir Samman, Shamasunder Acharya, Judy Luu, Chris Rissel, John Attia

**Affiliations:** 1 School of Medicine and Public Health, The University of Newcastle, Callaghan, NSW Australia; 2 Division of Medicine, Hunter New England Local Health District, New Lambton, NSW Australia; 3 Health Research and Translation and Population Health, Hunter New England Local Health District, New Lambton, NSW Australia; 4 Hunter Medical Research Institute, Newcastle, NSW Australia; 5 Diabetes Alliance, Hunter New England Local Health District, New Lambton, NSW Australia; 6 Department of Pharmacology, Faculty of Medicine, University of Colombo, Colombo, Sri Lanka; 7 Department of Physiology Faculty of Medicine, University of Colombo, Colombo, Sri Lanka; 8 School of Life and Environmental Sciences, University of Sydney, Sydney, NSW Australia; 9 The NSW Office of Preventive Health, South Western Sydney Local Health District, Liverpool Hospital, Liverpool, NSW Australia

**Keywords:** Zinc supplementation, Healthy lifestyle, Pre-diabetes, Australia, Adults

## Abstract

**Background:**

Diabetes is increasing in incidence, morbidity and treatment costs globally, hence prevention strategies need to be explored. Animal studies and some human data have shown that zinc can improve glycaemic control, but the impact of this effect in a pre-diabetic population remains uncertain. This study is designed to investigate whether zinc gluconate and lifestyle coaching can improve glucose handling and ultimately reduce diabetes incidence in an at-risk pre-diabetic population in Australia.

**Methods/design:**

The study will be a randomised, placebo-controlled, double-blind clinical trial. The study will be conducted at the Hunter New England Local Health District New South Wales (NSW), Australia. Pre-diabetic (haemoglobin A1c [HbA1c] 5.7–6.4) male and female participants (n = 410) aged 40–70 years will be recruited through the Diabetes Alliance Network, a collaboration of diabetes specialists and general practitioner practices. All participants will be given routine care to encourage healthy lifestyle changes using a telephone coaching service (Get Healthy Information and Coaching Service, NSW Health) and then randomised to receive a supplement, either zinc gluconate (equivalent to 30 mg of elemental zinc) or placebo of identical appearance for 12 months. The identity of the supplements will be blinded to both research personnel and the participants. Participants will be asked to complete medical, lifestyle and dietary surveys and will have baseline and final visits at their general practitioner practice. Primary outcomes will be HbA1c and insulin sensitivity collected at baseline and at 1, 6 and 12 months; secondary outcomes will include fasting blood glucose, fasting cholesterol, blood pressure and body mass index. The primary efficacy endpoint will be judged at 6 months.

**Discussion:**

This study will generate new evidence about the potential for health coaching, with or without zinc supplementation, to improve glucose handling and ultimately to reduce progression from pre-diabetes to diabetes.

**Trial registration:**

Australian and New Zealand Clinical Trials Registry, ACTRN12618001120268. Registered on 6 July 2018.

**Electronic supplementary material:**

The online version of this article (10.1186/s13063-019-3317-4) contains supplementary material, which is available to authorized users.

## Background

The global burden of diabetes (among adults aged 20–79 years) in 2015 was estimated at US 415 million and is expected to rise up to 642 million by 2040 [1–3]. The global health cost in 2015 was approximately US $673 billion with an expected 25.4% increased cost to US $802 billion in 2040 [2]. The number of deaths attributed to diabetes worldwide in 2015 was 5 million [2]. It is known that 90–95% of diabetes cases are type 2 diabetes mellitus (T2DM), which is a preventable and modifiable disease [1]. Pre-diabetes mellitus (PreDM) is a potentially reversible condition of impaired glucose handling. It is increasingly being diagnosed on the basis of a haemoglobin A1c (HbA1c) measurement, given the low coefficient of variation of this test [4]. The natural history of PreDM is variable; progression to T2DM occurs in ~ 25%; the abnormal glycaemic state remains in 50%; and reversion to normal glycaemic state occurs in 25% over 3–5 years [5]. People with PreDM are 5–15 times more likely to develop T2DM and are at increased risk of cardiovascular disease (CVD) [6]. The progression of disease through the pre-diabetic phase provides an opportunity to intervene to reduce or delay the onset of frank diabetes.

Interventions for PreDM are limited. Improving lifestyle factors, such as nutrition and physical activity, and maintenance of healthy weight have been found to reduce the progression from PreDM to T2DM by ~ 50%, although these changes take time to institute and are often difficult to maintain for the long term [7]. Despite the effectiveness of interventions to reduce such risk factors, the prevalence/incidence of obesity and PreDM continues to increase globally and in Australia [2, 8]. Given this trend, additional and complementary treatment options that will prevent, or at least delay, progression of PreDM are needed. Our intervention will involve the referral of all participants to an existing health promotion programme provided by the NSW State Health Department: the Get Healthy Information and Coaching Service [13]. The free telephone coaching service contacts participants on up to 13 occasions over the first 6 months of the study to encourage lifestyle improvements. In addition, participants will be randomised to receive either a zinc or placebo daily supplement.

### Rationale

It has long been known that insulin is stored with zinc in the secretory granules within the beta-cells of the pancreas [9]. Zinc participates in glucose handling in many ways:

- Zinc is involved in the synthesis, storage and secretion of monomeric insulin, as well as conversion to a dimeric form for storage and secretion as crystalline insulin [10, 11].
- Zinc is essential in insulin action and carbohydrate metabolism [9].
- Human islet amyloid polypeptide (HIAPP) is a hormone that is stored with insulin in the secretory granule and is released from the pancreatic beta-cells when blood glucose levels are elevated. Under normal conditions, zinc concentration in the beta-cells is the highest in the human body, and zinc plays a role in keeping glucose levels and HIAPP stabilised. When zinc status is low, HIAPP aggregates into amyloid fibres that in turn become cytotoxic to beta-cells [12].
- Zinc stimulates two different classes of zinc transporter which enhance glucose-stimulated insulin secretion [14].

Translation of this work to humans has occurred in three ways:

- Two separate meta-analyses of 25 different small-scale zinc supplementation trials have found a significant reduction in fasting blood glucose and HbA1c, as well as cholesterol, in people with PreDM and metabolic syndrome [15, 16].
- In prospective longitudinal cohorts, those with the highest quintile of dietary zinc intake have the lowest incidence of diabetes during follow-up [17, 18].
- A randomised controlled trial of zinc supplementation among people with PreDM reduced progression to diabetes from 25% to 11% over 1 year [1]. However, this study was completed in Sri Lanka, where the dietary intake may be different and the diabetes incidence is high [1, 3].

Given such findings, zinc supplementation may be a useful additional strategy to complement existing effective risk reduction interventions. Our study will add to the limited scientific literature and will be the first randomised placebo-controlled trial of its kind in Australia.

## Methods/design

### Aim

Our study is designed to help patients better manage their own health and prevent T2DM, using an existing and effective lifestyle intervention with and without a low-cost zinc supplement, in an Australian population of men and women with PreDM aged between 40 and 70 years.

### Objectives

#### Primary objective

- To determine if the Get Healthy Information and Coaching Service with zinc supplementation over 12 months improves glucose-handling parameters (HbA1c and insulin sensitivity [IS]) (Cohen’s *d* = 0.4) compared with the Get Healthy Information and Coaching Service with placebo supplementation.

#### Secondary objectives

- To determine if the Get Healthy Information and Coaching Service with zinc supplementation delays or prevents progression to diabetes mellitus compared with the Get Healthy Information and Coaching Service with placebo supplementation.
- To determine if the Get Healthy Information and Coaching Service with zinc supplementation improves CVD risk factors compared with the Get Healthy Information and Coaching Service with placebo supplementation.
- To determine if the Get Healthy Information and Coaching Service alone will improve glucose handling and CVD risk factors compared with each individual’s own baseline (within control group only).
- To determine if the Get Healthy Information and Coaching Service can be incorporated into a new model of care of patients with PreDM through general practitioner (GP) practices.
- To determine the cost-effectiveness of the new model of care (Get Healthy Information and Coaching Service and zinc supplementation) with early intervention.
- To determine the acceptability of daily zinc supplementation for patients with PreDM.

### Trial design

**Fig. 1:**
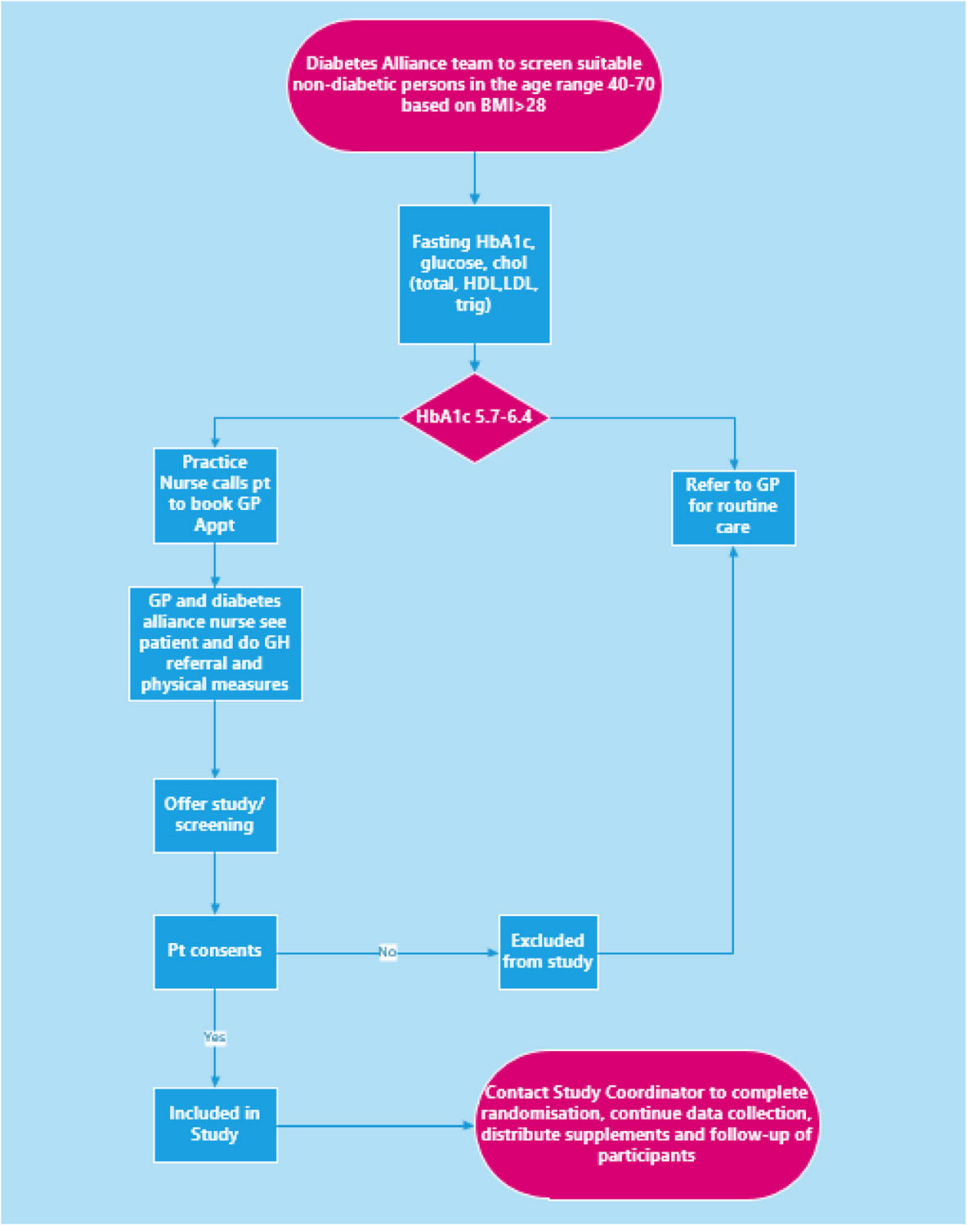
Participant timeline. Standard Protocol Items: Recommendations for Interventional Trials (SPIRIT) figure [20] (Additional file 1) for the study schedule of enrolment, interventions and assessments. Baseline assessments will occur at -t_1–0_ prior to allocation. Follow-up will occur at time points between t_1_ and t_5_ (1, 3, 6, 9 and 12 months after allocation). Adverse event monitoring will occur after allocation at t_2_, t_3,_ t_4_ and t_5_ (3, 6, 9 and 12 months); HbA1c, LDL, HDL, total cholesterol, triglycerides, fasting blood glucose and insulin will be collected at –t_1–0_, t_0_ (baseline), t_1_ (1 month) t_3_ (6 months) and t_5_ (12 months). Close-out assessments will occur at t_5_ (12 months)

The study will be a double-blind, randomised, parallel-group, placebo-controlled superiority trial. The allocation ratio will be 1:1 zinc supplementation and placebo, stratified by GP practice and biological sex (male and female). Participants will be followed up at 1 month, 6 months and 12 months. Both treatment arms will be referred to the Get Healthy Information and Coaching Service type 2 diabetes prevention program for the first 6 months of the trial (standard duration of the programme). The schematic of the trial recruitment is provided in Fig 1.

### Setting

Participants will be selected by the Diabetes Alliance team through GP practices in the Hunter New England Local Health District, NSW, Australia.

### Eligibility criteria

#### Inclusion criteria

To be eligible, men and women aged 40–70 years and with a body mass index (BMI) ≥ 28 kg/m^2^ will be screened for PreDM on the basis of one HbA1c measurement (5.7–6.4 mmol/L) as per the American Diabetes Association guidelines [19]. Potential participants must be willing to enrol in the Get Healthy Information and Coaching Service and not meet any of the exclusion criteria.

#### Exclusion criteria

Potential participants must not have any of the following: a diagnosis of diabetes; taking zinc, calcium or iron supplements; receiving a weight loss medicine or programme; alcohol intake > 20 g/day; impaired hepatic or renal function; lactating, pregnant or planning a family; or a history of any condition that the investigator may consider a contraindication to participation [1].

### Interventions

All participants will be enrolled in the Get Healthy Information and Coaching Service. This is a registered service run by the New South Wales state health department that provides up to 13 telephone calls over 6 months from allied health professionals who provide health information and support participants to achieve a better lifestyle; it focuses on diet, physical activity, smoking cessation and alcohol reduction [13].

The intervention group will receive a daily oral zinc gluconate capsule (30 mg of elemental zinc, provided by Blackmores, Sydney, Australia) to be consumed before breakfast for a period of 12 months. The placebo group will receive a cellulose capsule (appearance identical to the zinc capsule) to be taken similarly.

### Outcomes

#### Primary endpoints

The primary outcomes are fasting blood glucose (FBG) and HbA1c.

#### Secondary endpoints

The following are secondary endpoints:

- Progression to diabetes mellitus (based on FBG or HbA1c)
- CVD risk factors, including, for example, FBG, plasma cholesterol, body weight, BMI, smoking, alcohol consumption, dietary intake, physical activity and blood pressure
- Acceptability of the supplements, as shown by pill counts and adverse effects.

### Sample size

The sample size is based on an estimated mean difference in IS of 0.3 (with SD of 0.7, equal to Cohen’s *d* of 0.4) between the intervention and control groups. We will need 164 participants per group to reject the null hypothesis that the intervention and control group means are equal with probability (power) 0.9 and type I error probability (α) of 0.01. Allowing up to 20% loss to follow-up over 1 year, we will need to recruit a total of 410 participants.

### Screening and recruitment

Participants will be screened and selected through the Diabetes Alliance network. Men and women aged 40–70 years with a diagnosis of PreDM will be invited to participate in the study. Informed consent, baseline measures and questionnaire responses will be obtained.

### Randomisation and blinding

Research staff will randomise participants into the study using permuted blocks of sizes 4 and 6, stratified by GP practice and biological sex (male or female), programmed into a computerised research electronic data capture (REDCap) database [21]. The allocation will be concealed and study personnel, the participants and the statistician will be blinded. The study coordinator will send out the capsules (labelled as A or B) to participants according to the allocation at baseline and every 3 months until 1 year. The adjudicators for adverse events will also be blinded to the allocation. Unblinding will occur at the close of the study unless a participant wishes to withdraw from the study or the GP requests unblinding of a participant for medical reasons, in particular for the diagnosis of T2DM.

### Data collection methods

**Fig. 2:**
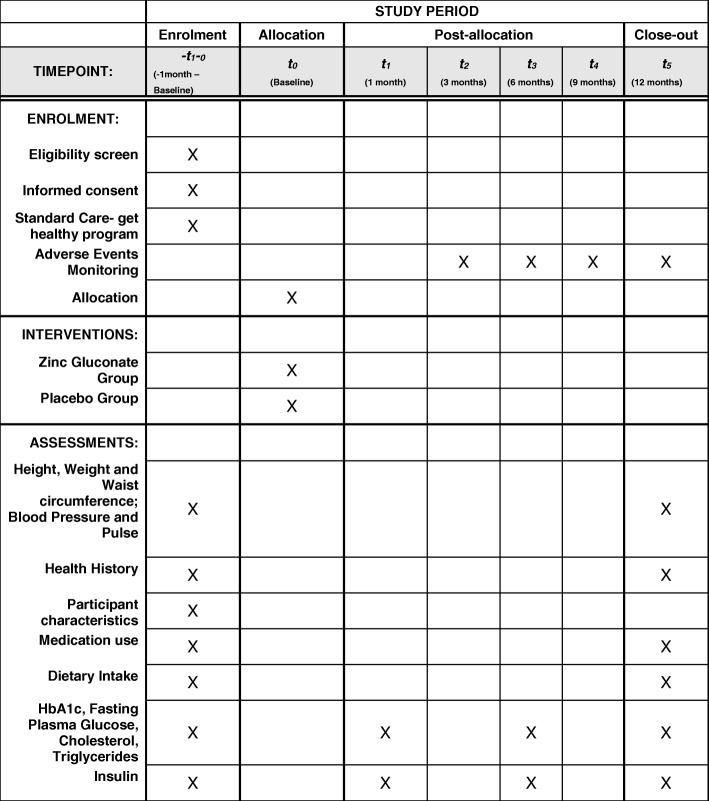
Trial design. Simplified schematic of trial design

The baseline and final visits will be at the participant’s designated GP practice. Anthropometric measures (height, weight and waist circumference), blood pressure and pulse will be collected by the diabetes nurse or the practice nurse at the GP practice. Questionnaires (medical history, medicine use, dietary intake and participant characteristics) will be sent to the participants’ nominated address. Participants can nominate to complete the majority of surveys online if they choose to do so. The adverse event questionnaire, blood request forms and new supply of supplement capsules will be mailed to participants every 3 months with a reply-paid envelope. Participants will return the questionnaire along with any remaining capsules previously supplied for research staff to record any adverse events and to record compliance using pill counts; participants will also undergo blood tests at their nearest pathology provider. The timeline for collection of the various data points is shown in Fig. 2.

### Data management

An online REDCap database will store all data collected. This database will be managed by an independent statistician.

### Statistical methods

Analyses will be undertaken in accordance with the Consolidated Standards of Reporting Trials (CONSORT) guidelines by an independent statistician blinded to treatment group allocation [22].

#### Effectiveness of zinc

##### Primary analysis

IS and HbA1c at baseline and at 1, 6 and 12 months will be plotted to assess response trajectories. We will fit a linear mixed model (LMM) with IS or HbA1c measured at 1, 6 and 12 months as the dependent variable and fixed effects for time (categorical predictor), group and time-by-group interaction terms. This will allow us to identify the time points at which maximal group differences are observed, although the primary outcomes will be judged at 6 months. A random intercept will be included to account for repeated measures on participants, and the model will be adjusted for baseline HbA1c or IS, age, sex, physical activity and diet. The LMM uses all available values for each participant and provides robust effect estimates assuming data are missing at random. All statistical analyses will observe the intention-to-treat principle. A significant difference on either of the primary outcomes will be sufficient to declare an effect, although we will also look at consistency in the direction of the secondary outcomes.

##### Secondary analyses

A similar LMM approach will be used to jointly analyse the data at baseline and 1, 6 and 12 months for insulin resistance, beta-cell function, lipids and other secondary outcomes across the zinc and control groups.

#### Effectiveness of Get Healthy T2DM Prevention Program

HbA1c values at baseline and at 1, 6 and 12 months will be plotted for the placebo group alone to allow us to describe the effect of the Get Healthy Information and Coaching Service without zinc supplementation. We will fit an LMM with fixed effects for time as a categorical predictor and HbA1c as the dependent variable. This will allow us to estimate the mean within-person pre-post differences due to lifestyle changes. A random intercept will be included to account for repeated measures on participants, and the model will be adjusted for baseline age, sex, physical activity and diet quality.

A similar LMM approach will be used to analyse IS and FBG and to jointly analyse the baseline and 12-month data for BMI, waist/hip ratio and blood pressure. In addition, completion rates for the Get Healthy Information and Coaching Service for those referred as part of the study will be benchmarked to current rates for those referred through other sources.

#### Cost-effectiveness of zinc and Get Healthy T2DM Prevention Program

##### Direct analyses

The cost-effectiveness of zinc supplementation will be assessed in a trial-based analysis. The analysis will adopt a modified societal perspective to account for health care system costs as well as patient costs which would be incurred if the zinc supplement were an out-of-pocket expense. The incremental cost-effectiveness ratio (ICER) will be calculated as the difference in average cost between the zinc supplementation group and the placebo group, divided by the difference in average FPG level. Uncertainty intervals for the incremental costs and ICER will be calculated using a non-parametric bootstrap procedure to reflect sampling uncertainty. Sensitivity analysis will examine imputation uncertainty, comparing the reference case results with the analysis of participants with complete data. Additional sensitivity analysis will involve variation in the market price of zinc capsules.

#### Modelled analyses

The results of the trial-based analysis will be extrapolated in a cost-consequence analysis comparing the health care resource use profiles for two cohorts over a 5-year period: those people with delayed progression to diabetes as a result of zinc supplementation in conjunction with effective lifestyle modification and those people who follow the natural history of diabetes progression.

A second modelled analysis will extrapolate the results from the trial-based analysis and transform the intermediate, primary trial outcome into a final, patient-relevant outcome: quality-adjusted life-years saved. The cost-utility analysis will model costs, expected cost savings and patient outcomes over the lifetime of a hypothetical cohort of patients with PreDM. Similar direct and modelled analyses will be performed for the evaluation of the Get Healthy Information and Coaching T2DM Prevention Program, looking at pre- and post-glycaemic markers.

#### Acceptability of zinc supplementation

Acceptability will be determined using compliance, assessed by quarterly pill counts. Average number of capsules taken divided by the theoretical maximum taken, over the four quarters, will be used to calculate percentage of apparent compliance [23].

### Data monitoring

An independent statistician will monitor the data collected and will review any adverse events which may require reporting.

### Harms

Participants will complete an “adverse events questionnaire” every 3 months to ascertain tolerance to the zinc or placebo. Any unexpected serious adverse events will be reported to the ethics committee and regulatory bodies.

### Auditing

Periodic audits will be attended by an independent statistician. The online database will have in-built structures to ensure data are entered appropriately and in a quality fashion.

## Discussion

This trial sets out to explore whether a new model of care for preventing diabetes in an at-risk Australian population can delay or prevent the onset of diabetes. The scientific literature has supporting evidence, but the sample sizes are small and follow-up duration is short in some instances. Also, the previous studies have been in Asian populations (Bangladesh and Sri Lanka) [1, 24] with differing physical and metabolic characteristics. Their dietary intakes are different, and they are more likely to have an underlying zinc deficiency compared with the overall Australian population. We do not expect a conversion rate from PreDM to diabetes to be as high as 25% over a 12-month period in the control group as observed in the Asian population group [1]. In the Australian population, progression from PreDM to diabetes can take from 7.5 years (females) to 9.5 years (males) or more in a similar age group [25]. However, any differences between treatment groups in the Australian population are important to note. Our study aims to support the current literature by recruiting 410 men and women with PreDM aged between 40 and 70 years who are residing in Australia who would benefit from lifestyle changes with or without zinc supplementation over a 12-month period. If the study proves successful, it will pave the way for a much larger, multi-centre clinical trial with frank diabetes as the endpoint.

## Trial status

This is protocol version 4, dated 25th September 2018 (ANZCTR registration number ACTRN 12618001120268). Recruitment commenced in November 2018 and is expected to be completed by July 2019.

## Additional file

Additional file 1: Standard Protocol Items: Recommendations for Interventional Trials (SPIRIT) checklist [26]. (DOC 122 kb)

## Abbreviations

ANZCTR: Australian and New Zealand Clinical Trials Registry
BMI: Body mass index
CVD: Cardiovascular disease
FBG: Fasting blood glucose
GH: Get Healthy Information and Coaching Service
GP: General practitioner
HbA1c: Haemoglobin A1c
HDL: High-density lipoprotein
HIAPP: Human islet amyloid polypeptide
ICER: Incremental cost-effectiveness ratio
IS: Insulin sensitivity
LDL: Low-density lipoprotein
LMM: Linear mixed model
PreDM: Pre-diabetes mellitus
REDcap: Research Electronic Data Capture
T2DM: Type 2 diabetes mellitus
